# Role of hedgehog signaling in the pathogenesis and therapy of heterotopic ossification

**DOI:** 10.3389/fcell.2024.1454058

**Published:** 2024-09-19

**Authors:** Yiran Pei, Fangzhou Liu, Yike Zhao, Hui Lin, Xiaoyan Huang

**Affiliations:** ^1^ The First Affiliated Hospital, Jiangxi Medical College, Nanchang University, Nanchang, Jiangxi, China; ^2^ Queen Mary School, Jiangxi Medical College, Nanchang University, Nanchang, Jiangxi Province, China; ^3^ Department of Pathophysiology, School of Basic Medical Sciences, Jiangxi Medical College, Nanchang University, Nanchang, Jiangxi, China

**Keywords:** hedgehog signaling, chondrocytes, osteoblasts, heterotopic ossification, therapeutics

## Abstract

Heterotopic ossification (HO) is a pathological process that generates ectopic bone in soft tissues. Hedgehog signaling (Hh signaling) is a signaling pathway that plays an important role in embryonic development and involves three ligands: sonic hedgehog (Shh), Indian hedgehog (Ihh) and desert hedgehog (Dhh). Hh signaling also has an important role in skeletal development. This paper discusses the effects of Hh signaling on the process of HO formation and describes several signaling molecules that are involved in Hh-mediated processes: parathyroid Hormone-Related Protein (PTHrP) and Fkbp10 mediate the expression of Hh during chondrogenesic differentiation. Extracellular signal-regulated kinase (ERK), GNAs and Yes-Associated Protein (YAP) interact with Hh signaling to play a role in osteogenic differentiation. Runt-Related Transcription Factor 2 (Runx2), Mohawk gene (Mkx) and bone morphogenetic protein (BMP) mediate Hh signaling during both chondrogenic and osteogenic differentiation. This paper also discusses possible therapeutic options for HO, lists several Hh inhibitors and explores whether they could serve as emerging targets for the treatment of HO.

## Introduction

Heterotopic ossification (HO) is defined as the development of mature lamellar bone in extraskeletal soft tissue. During World War I, HO inflicted additional damage on patients injured by explosions, which led to increased attention on the condition (Edwards and Clasper, 2015). HO can be caused by various factors, with the most common type being induced by inflammation following trauma. Additionally, HO can arise from genetic factors. Fibrodysplasia ossificans progressiva (FOP) and progressive osseous heteroplasia (POH) are two forms of hereditary HO caused by different genetic defects.

### Introduction of HO: tHO, FOP and POH

Heterotopic ossification (HO) can occur in various conditions, including traumatic heterotopic ossification (tHO), fibrodysplasia ossificans progressiva (FOP), and progressive osseous heteroplasia (POH). The primary pathological mechanism underlying tHO is heterotopic endochondral ossification (HEO), where mesenchymal progenitor cells aggregate, proliferate, and differentiate into chondrocytes, which are later replaced by osteoblasts (Mackie et al., 2011), partly mimicking physiological bone formation (Culbert et al., 2014). Many cytokines and growth factors, including bone morphogenetic protein (BMP), transforming growth factor-β1 (TGF-β1), and vascular endothelial growth factor (VEGF) are involved in the molecular mechanism of tHO by promoting the differentiation of mesenchymal stem cells (MSCs) (Huang et al., 2021). Additionally, an increasing number of studies have shown the involvement of Hh signaling in formation of tHO (Li et al., 2022).

FOP, a rare autosomal dominant disorder, is specifically associated with HEO (Acosta and Fernández, 2021). The genetic basis for FOP is linked to heterozygous mutations in the activin receptor A type I (ACVR1 or ALK2), with the R206H mutation present in over 95% of patients (Ravazzolo and Bocciardi, 2021). These ACVR1 mutations are crucial for ectopic bone formation in FOP and form the basis of the disorder’s pathogenesis. Normally, BMP activates Smad1/5 phosphorylation by binding to the BMP type I receptor ACVR1 (Lin et al., 2019). Following the R206H mutation, a cytokine known as activin A can be stimulated by injured soft tissue (Chen et al., 2011; Phillips et al., 2009), which aberrantly induces Smad1/5 phosphorylation and enhances BMP signaling, ultimately significantly promoting chondrogenic and osteogenic differentiation.

POH is a hereditary condition characterized by progressive extraskeletal bone growth without an association with trauma or inflammation. Unlike FOP, POH exhibits no congenital skeletal deformities or inflammatory features, and the occurrence of intramembranous ossification rather than endochondral ossification. In POH, the process of chondrocyte formation is bypassed, and osteoblasts are formed directly (Kaplan and Shore, 2000). The pathogenesis of POH is driven by heterozygous inactivating mutations in the GNAS gene. GNAS, located on human chromosome 20, encodes the α-subunit of the stimulatory G protein, which is a signaling protein crucial for hormonal actions (Bastepe, 2018). Although the precise mechanism by which GNAS mutations lead to heterotopic ossification remains unclear, there is evidence suggesting involvement of the Hh signaling pathway (Regard et al., 2013).

### Hh signaling pathway

**FIGURE 1:**
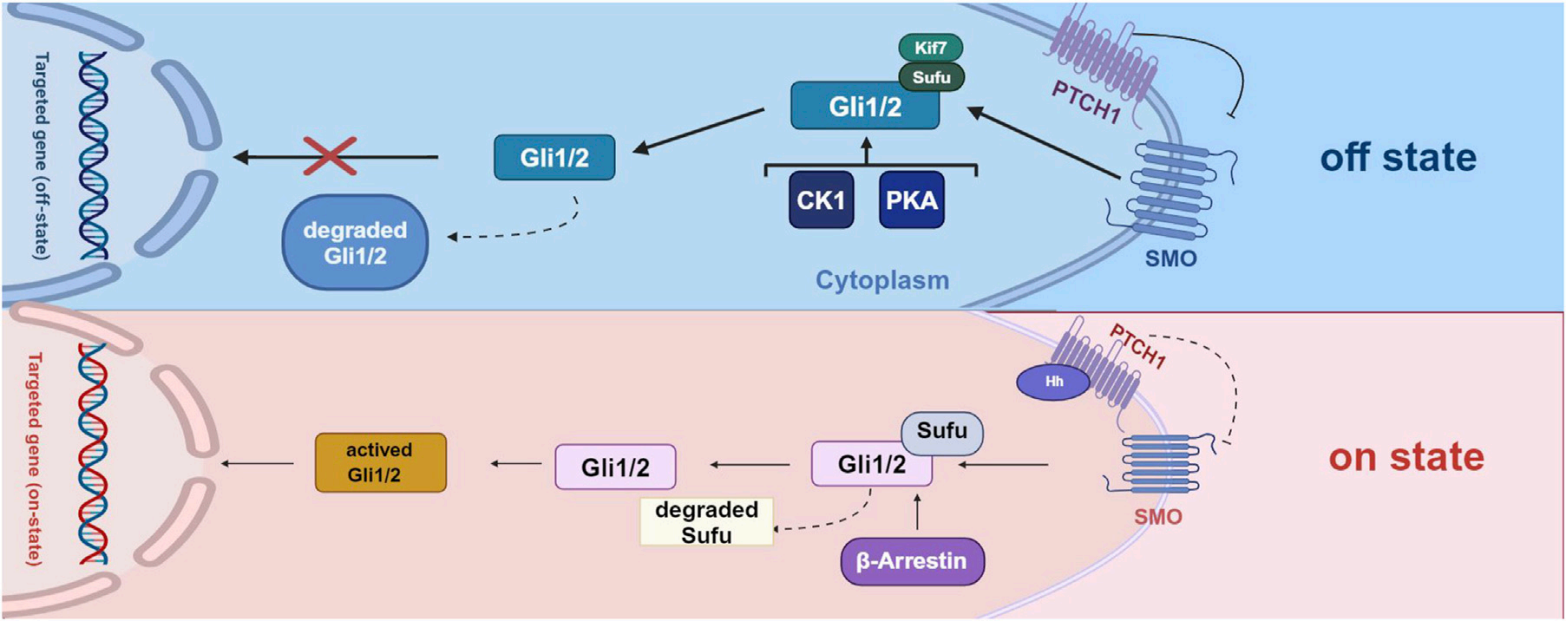
The process of activation of Hh signaling pathway and the comparison between two states.

Hedgehog (Hh) signaling occurs through three distinct types of signaling: Sonic hedgehog (Shh), Indian hedgehog (Ihh), and desert hedgehog (Dhh) (Skoda et al., 2018). Hh signaling begins with the binding of Hh ligands to the cell surface receptor Patched 1 (PTCH1), which results in the release of the cell surface protein Smoothened (SMO) from repression in the primary cilium, a subcellular protrusion extending from the apical surface of mammalian cells. In the absence of Hh ligands, PTCH1 suppresses SMO activity. Upon Hh ligand binding, SMO is activated, initiating downstream signal transduction by degrading Sufu (Suppressor of Fused) and activating proteins, including Gli1, Gli2, and Gli3. Several other cytokines, such as casein kinase 1 (CK1), protein kinase A (PKA), and β-arrestin are also involved in regulating signals downstream of Hh signaling (Fang et al., 2022) (Figure 1). Recently, Hh has been shown to play a role in cartilage and bone development (Cong et al., 2021). Dysregulation of Hh signaling can disrupt musculoskeletal tissue homeostasis and lead to a range of skeletal disorders, including HO (Feng et al., 2020).

In the off state, the Hh ligand does not bind to PTCH1, leading to the repression of SMO expression. Consequently, Gli1 and Gli2 are ultimately degraded and cannot function as transcription factors to mediate gene expression. In the on state, when the Hh ligand binds to PTCH1, the repressive effect of PTCH1 is alleviated, resulting in the degradation of Sufu factors and the activation of Gli1/2 factors. These activated factors regulate downstream signaling by initiating the transcription of target genes. Additionally, various other factors, including protein kinase A (PKA), casein kinase 1 (CK1), and β-arrestin, also play a role in the downstream regulation of Hh signaling (Created with BioRender.com).

### Hedgehog signaling in three types of HO

As mentioned above, the chondrogenesis and osteogenesis of mesenchymal stem cells (MSCs) are crucial steps in the process of traumatic heterotopic ossification (tHO). In the formation of tHO, Hedgehog (Hh) signaling can act directly on MSCs to regulate chondrogenic and osteogenic differentiation. The upregulation of Hh signaling promotes ectopic chondrogenesis and osteogenesis in traumatized tissues, which in turn facilitates the generation of heterotopic ossification (HO). Hh also plays a role in the process of HO formation by regulating the activity of reactive oxygen species (ROS). Research has demonstrated that ROS affect chondrogenic and osteogenic differentiation differently across various disease models: for MSCs, the upregulation of ROS impairs osteogenic differentiation while stimulating chondrogenesis (Li et al., 2017); conversely, in tendon-derived stem cells (TDSCs), high levels of ROS promote both osteogenic and chondrogenic differentiation and facilitate HO formation. Hh can modulate the antioxidant pathway in TDSCs to influence the activity of ROS, thereby participating in the production of tendon-derived HO (Ruiz-Heiland et al., 2012).

The formation of fibrodysplasia ossificans progressiva (FOP) is associated with the expression of Sonic hedgehog (Shh) and Indian hedgehog (Ihh). Mutations in the ACVR1 gene have been shown to induce the expression of Shh and Ihh, promoting the differentiation of osteoblasts. This process involves a positive feedback loop between Shh and Yes-associated protein (YAP) (Cong et al., 2021). However, the precise mechanisms by which Hh signaling is involved in FOP remain unclear and require further investigation.

Hedgehog (Hh) signaling plays a crucial role in intramembranous ossification diseases, particularly in progressive osseous heteroplasia (POH), which is caused by heterozygous null mutations in the GNAS (Pignolo et al., 2015). Enhanced Hh signaling mediates the downstream effects of GNAS mutations. The specific underlying mechanisms are described below.

### Hedgehog activity in chondrogenic differentiation

Chondrogenic differentiation is a crucial step in endochondral ossification. Initially, undifferentiated mesenchymal stem cells (MSCs) accumulate in large numbers, differentiate into chondrocytes, and proliferate extensively starting from the center. These chondrocytes further differentiate into hypertrophic chondrocytes, forming an avascular cartilaginous template surrounded by a perichondrium, and secrete a specialized extracellular matrix (Yang et al., 2014). Indian hedgehog (Ihh) is the primary signaling molecule within the Hedgehog (Hh) family that is involved in this process (St-Jacques et al., 1999). The Hh signaling target genes Patched 1 (Ptch1) and Gli1 are expressed in both proliferating chondrocytes and perichondrocytes, promoting chondrocyte proliferation and differentiation (Ohba, 2016). Additionally, other signaling molecules are also involved in the Hh-mediated regulation of chondrogenic differentiation.

### Fkbp10

**FIGURE 2:**
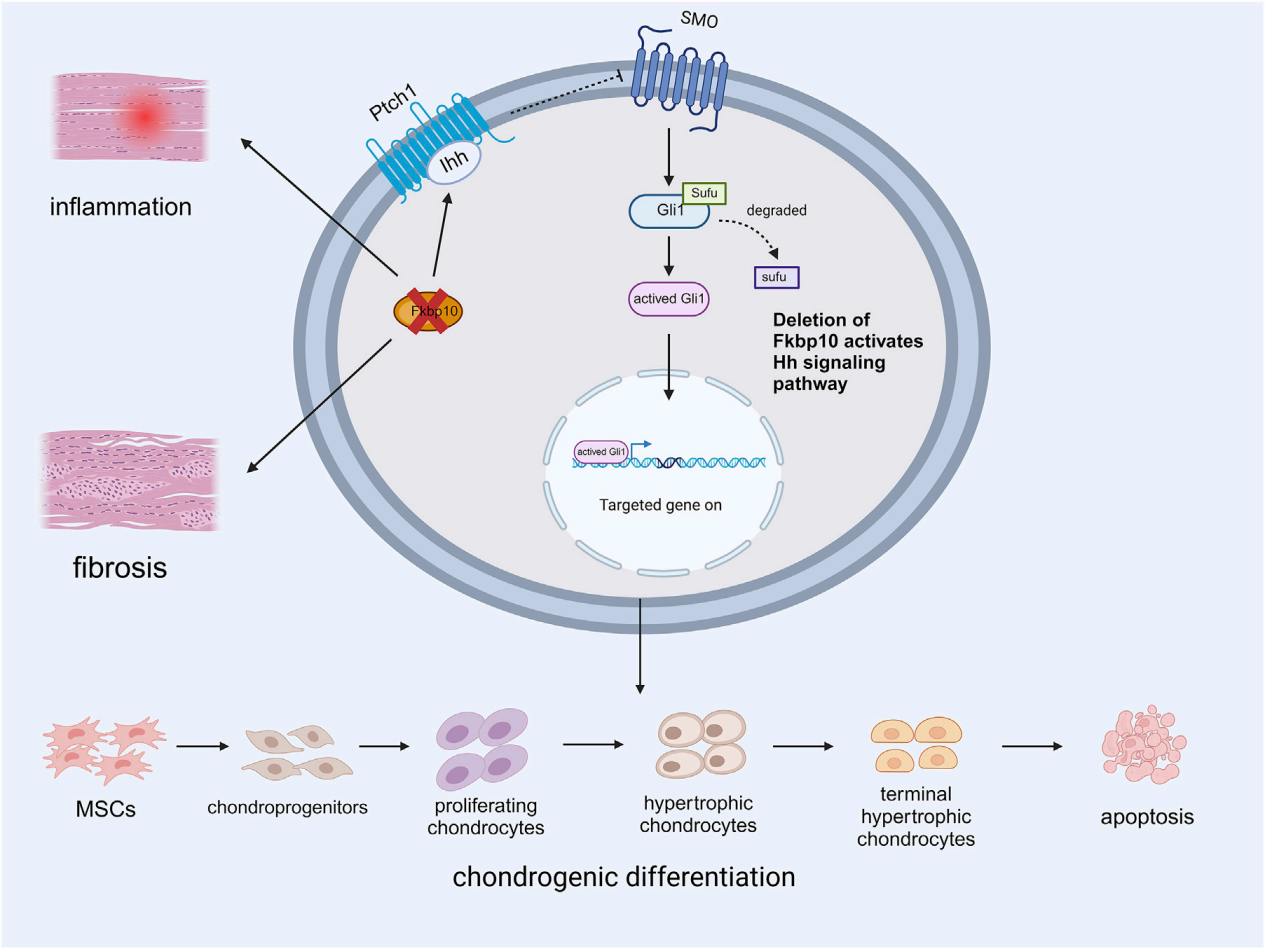
Expression of Ihh Mediated by Deletion of Fkbp10 during Chondrogenic differentiation.

Fkbp10 is a member of the immunophilin family and frequently exhibits a repeat in the peptidylprolyl isomerase (PPIase) domain (Davis et al., 1998). Fkbp10 is crucial for the normal development of tendons and ligaments. Experimental results revealed that the absence of Fkbp10 in the tendons and ligaments of postnatal mice led to the upregulation of fibrosis and inflammatory genes, resulting in joint malformation and heterotopic ossification (HO). Deletion of Fkbp10 also affects the expression of the Hedgehog (Hh) signaling pathway. Immunohistochemistry showed high levels of Indian hedgehog (Ihh) in areas of ectopic chondrogenesis, with the expression level of Gli1 correlating with that of Ihh. Subsequent knockout of Smoothened (SMO) reduced ectopic cartilage formation in mice, suggesting that inhibition of the Hh signaling pathway mitigates HO caused by Fkbp10 deletion. These studies confirmed that the loss of Fkbp10 is associated with ectopic chondrogenesis in connective tissue, followed by joint deformities and HO (Lim et al., 2021). High levels of Ihh and Gli1 were detected in tissues surrounding the ectopic chondrogenesis sites, indicating that Fkbp10 mutation upregulates the transduction of the Ihh signaling pathway to promote chondrogenesis in HO(17) (Figure 2). However, the exact mechanism by which Hh signaling is dysregulated in Fkbp10 mutants remains unknown, and further research is necessary.

The deletion of Fkbp10 promotes inflammation and fibrosis in tendons and ligaments while enhancing the binding of the Ihh ligand to PTCH1. This abolishes the inhibitory effect of PTCH1 on SMO, and the subsequent degradation of SUFU leads to the activation of Gli1. This activation promotes the aberrant differentiation of tendon-derived MSCs into chondrocytes by facilitating the expression of relevant genes (Created with BioRender.com).

### PTHrP and Runx2

**FIGURE 3:**
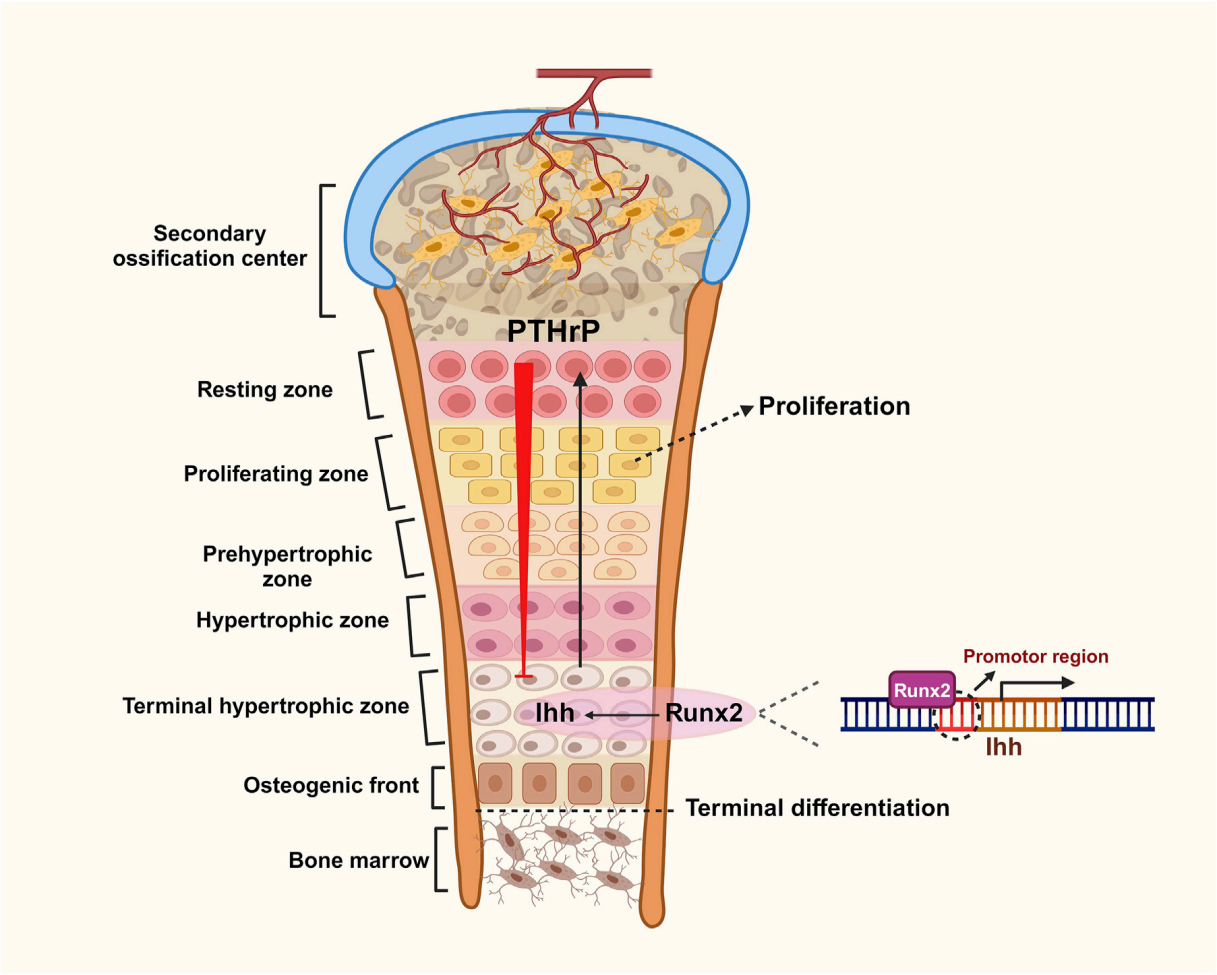
Role of negative feedback loops between Ihh, PTHrP and Runx2 in chondrogenic differentiation.

As mentioned earlier, Indian hedgehog (Ihh), a member of the hedgehog family, plays a crucial role in both physiological and pathological skeletal development by mediating the proliferation and differentiation of chondrocytes. Two key signaling molecules involved in this process are runt-related transcription factor 2 (Runx2) and parathyroid hormone-related peptide (PTHrP) (Kronenberg, 2003). Runx2 is an essential transcription factor for chondrocyte differentiation. It induces Ihh expression, which organizes chondrocyte maturation and proliferation. Research shows that mice lacking Runx2 fail to mature chondrocytes and do not express Ihh. However, after the implantation of adenoviruses containing Runx2, Ihh expression is restored, highlighting Runx2’s critical role in chondrocyte maturation (Komori, 2018; Komori, 2019). In the growth plate, Ihh promotes the proliferation and differentiation of prehypertrophic chondrocytes and stimulates the release of PTHrP from nearby chondrocytes and perichondrocytes. PTHrP acts on PTH/PTHrP receptors (PPRs) to maintain proliferative chondrocytes while inhibiting Runx2 and Ihh synthesis, thus suppressing chondrocyte proliferation and differentiation. When the source of PTHrP is sufficiently distant, inhibition of Runx2 and Ihh wanes, allowing Runx2 to induce Ihh synthesis again (St-Jacques et al., 1999). These mechanisms illustrate how Runx2, Ihh, and PTHrP collaborate to influence chondrocyte fate through a negative feedback loop that regulates terminal chondrocyte differentiation (Fang et al., 2022) (Figure 3). Additionally, Ihh independently activates BMP expression to promote chondrocyte differentiation without involving PTHrP (Mak et al., 2008).

The growth plate is comprised of five distinct zones: the resting zone, proliferating zone, prehypertrophic zone, hypertrophic zone, and terminal hypertrophic zone. During cartilage proliferation, Runx2 directly binds to the promoter region of the Ihh gene, promoting its transcription in prehypertrophic chondrocytes. This action stimulates the proliferation and differentiation of chondrocytes and induces the release of PTHrP from the zone of resting cartilage at the ends of the longitudinal valleys. PTHrP conversely inhibits the stimulatory effect of Runx2 on Ihh through a negative feedback loop, which collectively regulates cartilage proliferation (Created with BioRender.com).

### Hedgehog activity in osteogenic differentiation

Osteogenic differentiation varies between endochondral and intramembranous ossification. In endochondral ossification, osteogenic differentiation occurs after cartilage differentiation, with the first osteoblasts arising from the precursors of the chondrocyte membrane, giving rise to the bone collar. This process is followed by the vascular delivery of mesenchymal stem cells (MSCs) through the bone collar, which then differentiate into osteoblast progenitor cells that further develop into osteoblasts (Yang et al., 2014). In contrast, during intramembranous ossification, osteoblasts directly differentiate from MSCs into osteoprogenitor cells, which subsequently differentiate into osteoblasts (Ohba, 2016; Galea et al., 2021). Hedgehog (Hh) signaling molecules are also involved in the regulation of osteogenic differentiation. In addition to negatively regulating chondrocyte differentiation through feedback mechanisms involving parathyroid hormone-related peptide (PTHrP) and Runx2, Indian hedgehog (Ihh) plays a significant role in osteogenic differentiation. Osteoblasts first appear in the area surrounding the cartilage adjacent to prehypertrophic and hypertrophic chondrocytes. In hypertrophic chondrocytes, Ihh signaling is activated, promoting the expression of Gli2. The upregulation of Ihh and Gli2 enhances alkaline phosphatase (ALP) and osteocalcin (OCN) activity, suggesting that the activation of Ihh and Gli2 promotes osteoblast differentiation. High expression levels of Ihh in hypertrophic chondrocytes are essential for osteoblast differentiation (Ohba, 2016). In addition to Ihh, Sonic hedgehog (Shh) is also activated to promote osteogenic differentiation. Osteogenic differentiation markers such as ALP, Osterix (OSX), and bone sialoprotein (BSP) are secreted by osteoprogenitor cells and can be detected as evidence that osteogenic differentiation has occurred (Zhou et al., 2022).

### Runx2

**FIGURE 4:**
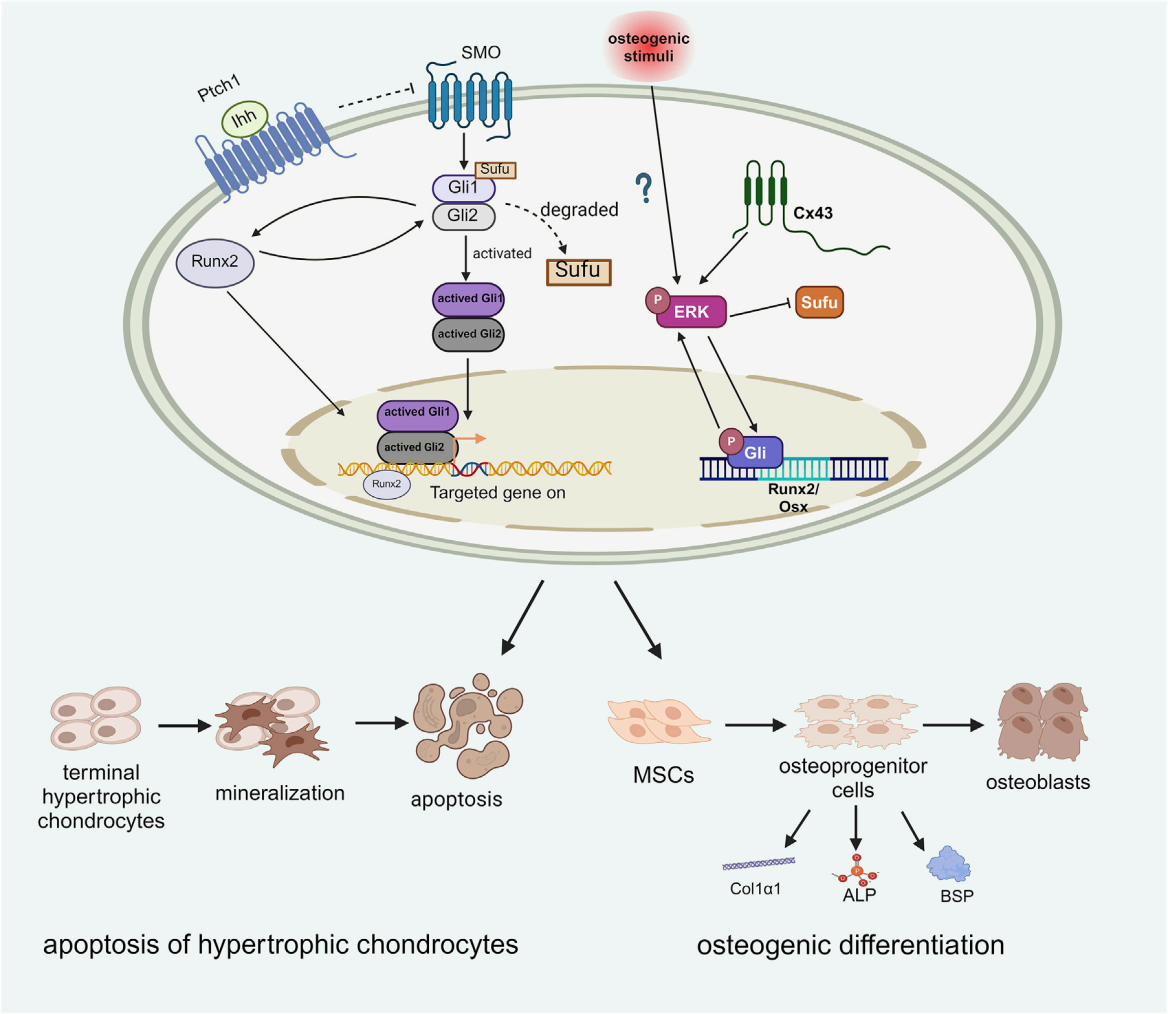
The role of Runx2 and ERK in osteogenic differentiation.

Runx2 also regulates osteoblast differentiation and Indian hedgehog (Ihh) is still necessary for Runx2 expression during this process. Ihh activation promotes Gli2 expression, which induces Runx2, and Runx2 subsequently activates Ptch1 and Gli1 to further enhance Ihh signaling. This mutual promotion between Runx2 and Ihh facilitates osteoblast differentiation, controls the mineralization of terminal hypertrophic chondrocytes, converts MSCs into osteoblast precursors, and regulates osteoblast markers such as type I collagen α1 chain (Col1α1), ALP, and BSP, independently of PTHrP. (Yoshida et al., 2004; Shimoyama et al., 2007). This indicates that, unlike PTHrP, both Runx2 and Ihh promote osteoblast differentiation (Komori, 2018). The conversion of chondrocytes into osteoblasts is vital for endochondral ossification (Yang et al., 2014) (Figure 4). However, Tu et al. recently reported that enhancing Runx2 expression in skeletal cells did not reverse bone formation in mice lacking Ihh, suggesting that Runx2 is not essential and that other factors mediate Ihh signaling in osteoblast differentiation. (Tu et al., 2012).

## ERK

Extracellular signal-regulated kinase (ERK) signaling has been shown to regulate the immune system and plays significant roles in osteogenic processes (Zhang et al., 2022; Barruet et al., 2018; Mishra et al., 2020; Li et al., 2021). Similarly, Hedgehog (Hh) signaling promotes mesenchymal stem cell (MSC) differentiation towards osteogenesis and aids in fracture repair (Williams et al., 2018). A study indicated that Hh signaling interacts with ERK, influencing abnormal osteogenic differentiation (Williams et al., 2018). In osteoprogenitors from heterotopic ossification (HO) patients, elevated levels of phosphorylated ERK (P-ERK) and Gli1 were observed following osteogenic stimuli. Inhibition of Hh and ERK signaling through cyclopamine and U0126 resulted in decreased P-ERK and Gli1 levels, highlighting their critical roles in osteogenesis. Additionally, ERK signaling has been proposed to be involved in HO production in murine soft tissues downstream of Connexin 43 (Cx43) (Cong et al., 2021; Kan et al., 2019). Cx43, expressed by osteocytes, osteoblasts and osteoclasts, has been observed in elbow HO patients at the clinical level and activates ERK signaling, contributing to traumatic HO formation and elevated osteogenesis-associated markers, including BSP, Col1α1 and ALP markers were noted in elbow HO patients. Furthermore, HO patients with strong Cx43 expression tended to have a greater recurrence rate after surgery, while a similar prognosis was also observed for HO patients with high p-ERK levels. These findings support the interactions among ERK, Cx43, and Hh signaling in osteogenic commitment (Figure 4) and underline the need for further studies on their crosstalk for potential HO therapies (Tu et al., 2016).

Runx2 and ERK are both key factors that influence Hh signaling to mediate the process of osteogenic differentiation. Runx2 interacts with Ihh to promote the apoptosis of terminal hypertrophic chondrocytes and facilitate osteogenic differentiation. The effect of ERK on Hh signaling is regulated by Cx43, which undergoes bidirectional positive coactivation with Gli. Gli serves as a TF that promotes the expression of osteogenic genes, including Runx2 and Osx. Additionally, Cx43 may act as an osteogenic stimulus that activates ERK through phosphorylation. Phosphorylated ERK can be activated during HO formation; the active form of ERK can recruit additional ERK molecules to converge with the Gli pathway, thereby promoting osteogenic development (Created with BioRender.com).

## GNAs and YAP

**FIGURE 5:**
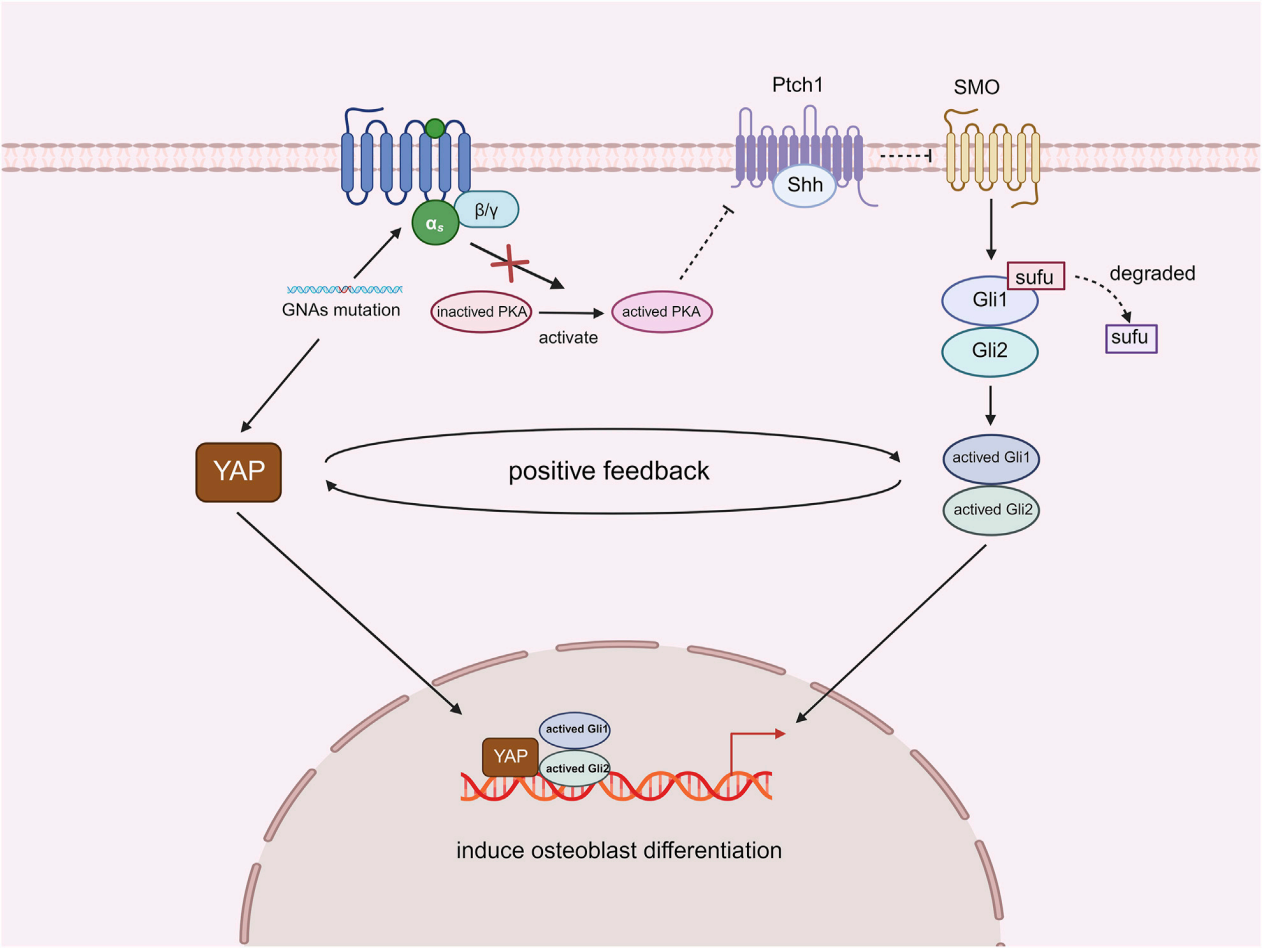
Effect of YAP and Shh interactions on HO after deletion of GNAs.

As mentioned above, GNAS is a gene that encodes the α-subunit of the stimulatory G protein. GNAS physiologically stimulates protein kinase A (PKA), which can inhibit Hedgehog (Hh) signaling transduction by regulating the activation of Gli. This indicates that a significant function of GNAS *in vivo* is the inhibition of the Hh signaling pathway (Regard et al., 2013). Deletion of GNAS leads to an upregulation of Hh signaling. Experimental analyses have shown that sonic hedgehog (Shh) is the Hh ligand predominantly activated by the deletion of GNAS. The activation of Shh signaling is both necessary and sufficient for the pathological mechanism of polysyndactyly associated with osteochondromas (POH) (Regard et al., 2013). In addition to Hh signaling, another important signaling molecule, yes-associated protein (YAP), is involved in the formation of POH. YAP is a transcription factor associated with the Hippo signaling pathway and serves as a crucial regulator of neoplastic and developmental events (Guo et al., 2004; Pan, 2010). The loss of GNAS also activates YAP transcription, and both YAP and Shh are located downstream of the GNAS signaling pathway. These two molecules can enhance each other’s expression and influence heterotopic ossification (HO) formation. RNA-seq and PCR analyses of GNAS-deficient cells confirmed an increase in YAP transcriptional activity, suggesting that the loss of GNAS activates YAP expression. Comparatively, the absence of GNAS led to a dramatic alleviation of HO symptoms when YAP was lost. Complete depletion of YAP entirely eliminated HO and significantly decreased Shh protein synthesis due to YAP deletion. These findings indicate that in the context of GNAS deficiency, YAP activation is essential for inducing Shh expression, which in turn causes HO and promotes osteoblast differentiation. Additionally, Shh activates YAP, and Shh deletion reduces YAP expression in mesenchymal cells. Recombinant Shh increased the expression of YAP target genes, osteoblast markers, and Hh signaling targets, demonstrating that Shh and YAP work together to create a positive feedback loop that encourages osteoblast development (Figure 5). The self-amplifying and self-propagating cycle of YAP and Shh is a key mechanism for bone formation in POH. This cycle also contributes to the formation of fibrodysplasia ossificans progressiva (FOP) and is necessary for HO. Therefore, the interaction between YAP and Shh may require increased attention as a core step in the pathogenesis of HO to explore new therapeutic strategies (Cong et al., 2021).

Mutations in GNA genes activate YAP, while the inactivation of PKA results in the absence of further inhibition of Shh signaling. Both YAP and Shh signaling function as transcription factors that promote osteoblast differentiation by mediating the expression of relevant genes. Furthermore, these two factors can establish a positive feedback loop that enhances the process of intramembranous ossification and exacerbates the progression of POH (Created with BioRender.com).

### Signaling molecules involved in both Hh-mediated chondrogenic and osteogenic differentiation

There are also signaling molecules that are involved not only in the process of Hh-mediated heterotopic ossification but also significantly influence both chondrogenic and osteogenic differentiation.

#### Sufu

Activated Hedgehog (Hh) signaling can drive heterotopic ossification (HO) in a cell-autonomous manner. It is important to note that HO produced in this manner is not part of the hereditary HO discussed in this paper. Sufu acts as a fusion repressor in the Hh pathway, functioning between the Smoothened (SMO) and Gli transcription factors (Tukachinsky et al., 2010). Sufu inhibits Gli proteins by directly binding to them, thereby preventing their translocation into the nucleus (Tukachinsky et al., 2010). This regulation is crucial for the stable expression of Gli, thus maintaining accurate Hh signaling (Figure 1). The deletion of Sufu is likely associated with the formation of HO. Feng et al. utilized Cathepsin K (Ctsk)-expressing mice to investigate the effects of Sufu on HO. Ctsk is a cysteine protease released by osteoblasts, and the Ctsk promoter serves as a marker for osteoclasts, perichondrial progenitors, and periosteum stem/progenitor cells in the perichondrial groove. The Ctsk-Cre-tagged tendon-derived progenitor cells (TDPCs) expressed the tendon marker scleraxis (SCX) in the middle of the tendon, and these subpopulations exhibited strong differentiation potential in response to Hh signaling. The experimental results indicated that Sufu deficiency leads to pronounced chondrogenic and osteogenic differentiation, which is associated with the dysregulation of Gli1 and Gli2 in classical Hh signaling. A lack of Sufu causes dysregulation of SMO, which in turn affects the mobilization of the Sufu zinc finger transcription factor Gli2. Gli2 translocates from the cilium to the nucleus, subsequently activating the Gli1 promoter. Thereafter, Gli1 and Gli2 directly stimulate the transcription of Hh-targeted genes, resulting in HO.Experiments have demonstrated that the deletion of Gli1 and Gli2 in Ctsk-CKO mice significantly reduces the extent of HO in the ligaments surrounding the ankle joints and Achilles tendons. This finding suggests that a certain level of Hh signaling activity is necessary for maintaining progenitors in ligaments, tendons, and the perichondrium. In summary, enhancing Hh signaling by deleting Sufu in Ctsk-expressing cells can induce HO in tendons and ligaments. Notably, Sufu deletion leads to activation of Hh signaling independently of Hh ligands, and there are differences between ligand-dependent Hh signaling and cell-autonomous Hh signaling induced by sufu deletion (Varjosalo and Taipale, 2008; Yin et al., 2019). Additionally, JQ1, a small-molecule suppressor, can inhibit the transcription of Gli1 and Gli2, thereby restraining the expression of Hh-targeted genes. Given JQ1’s ability to inhibit HO triggered by the upregulation of Hh signaling, it may represent a new therapeutic approach for treating endochondral HO in tendons and ligaments (Feng et al., 2020).

#### BMP

**FIGURE 6:**
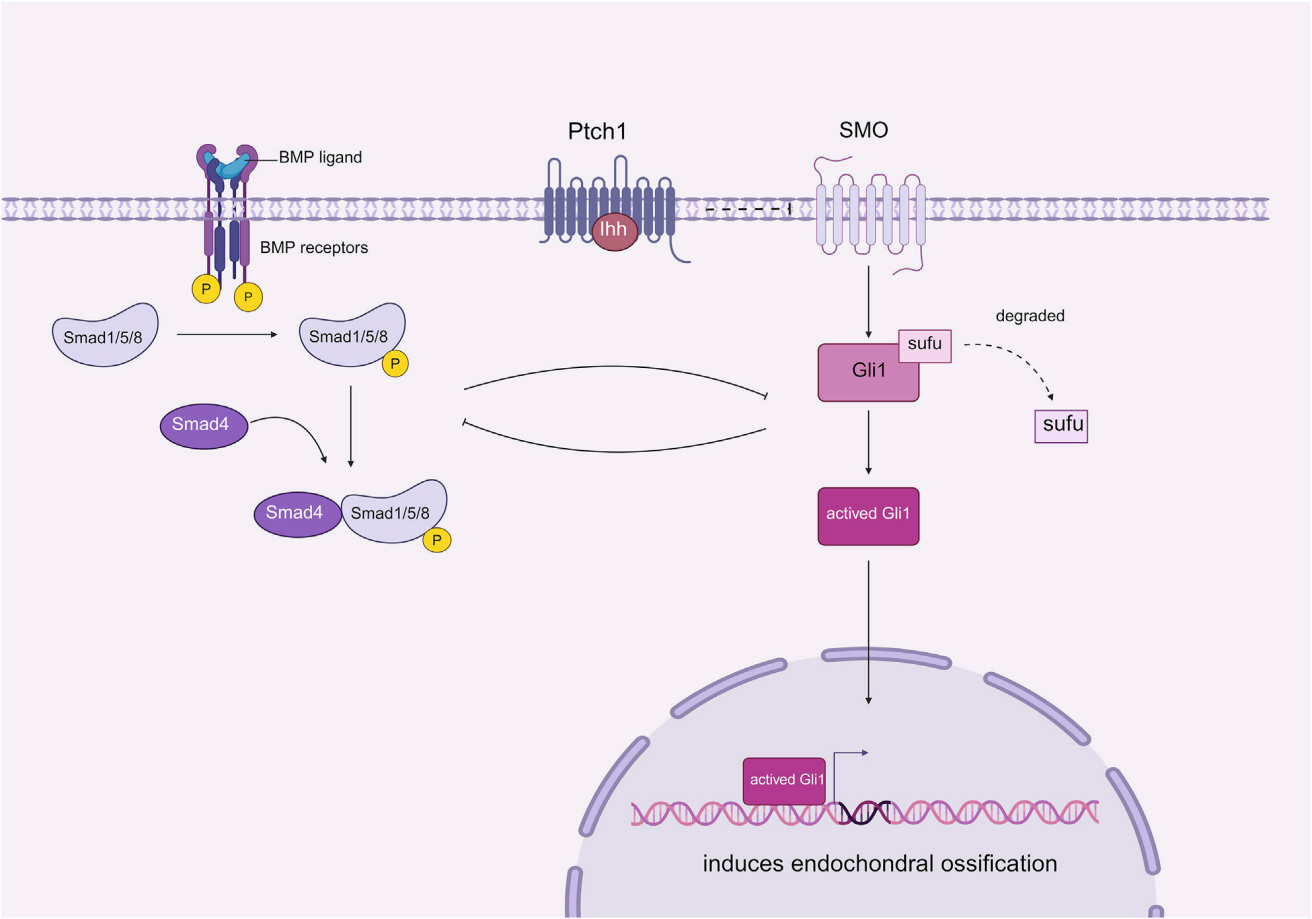
Effect of mutual inhibition between BMP and downstream Ihh on HO.

Bone morphogenetic protein (BMP) is a member of the transforming growth factor-beta (TGF-β) family. BMPs play a crucial role in tissue homeostasis and development, including the regulation of bone homeostasis. BMP signaling stimulates the condensation of mesenchymal stem cells (MSCs), promoting their proliferation and differentiation into chondrocytes and osteocytes (Wang et al., 2017). BMP signaling, which is the most widely studied pathway involved in heterotopic ossification (HO), has been shown to interact with the Hedgehog (Hh) signaling pathway to regulate the process of HO(48). Studies conducted by Chen et al. demonstrated that the BMP signaling gradient is nearly inversely correlated with Hh signaling elements such as Indian hedgehog (Ihh), Patched, and Gli1. These findings suggest that the MSC niche and the subsequent HO process may be co-regulated by Hh and BMP signaling, possibly through mutual inhibition (Kan et al., 2018b). The authors inferred that Hh signaling acts as a downstream mediator of BMP signaling and that the two pathways can partially inhibit each other (Figure 6). To test their hypothesis, Gli1-null mice, which highly express BMP signaling, were generated as experimental groups, while normal adult mice served as control groups. The HO in both groups was compared following trauma induction. The results indicated that HO was significantly suppressed in Nse-BMP4 mice, and the typical BMP signaling gradient was not observed, which provided strong evidence that Hh and BMP signaling can mediate the formation of HO through mutual inhibition. However, although HO was inhibited in Gli1-null mice, different stages of the process were affected to varying degrees, suggesting that other unknown factors may be involved in mediating this process (Kan et al., 2019). Nevertheless, the regulatory mechanism of BMP in the formation of fibrodysplasia ossificans progressiva (FOP) remains unclear and requires further study (Kan et al., 2018a).

Ihh signaling occurs downstream of BMP signaling, where BMP plays a role in suppressing the expression of Ihh, thereby facilitating the process of endochondral ossification. Interestingly, Ihh can also inhibit BMP activity simultaneously. This mutual inhibition between BMP and Ihh significantly influences the development of tHO (Created with BioRender.com).

#### Mkx

**FIGURE 7:**
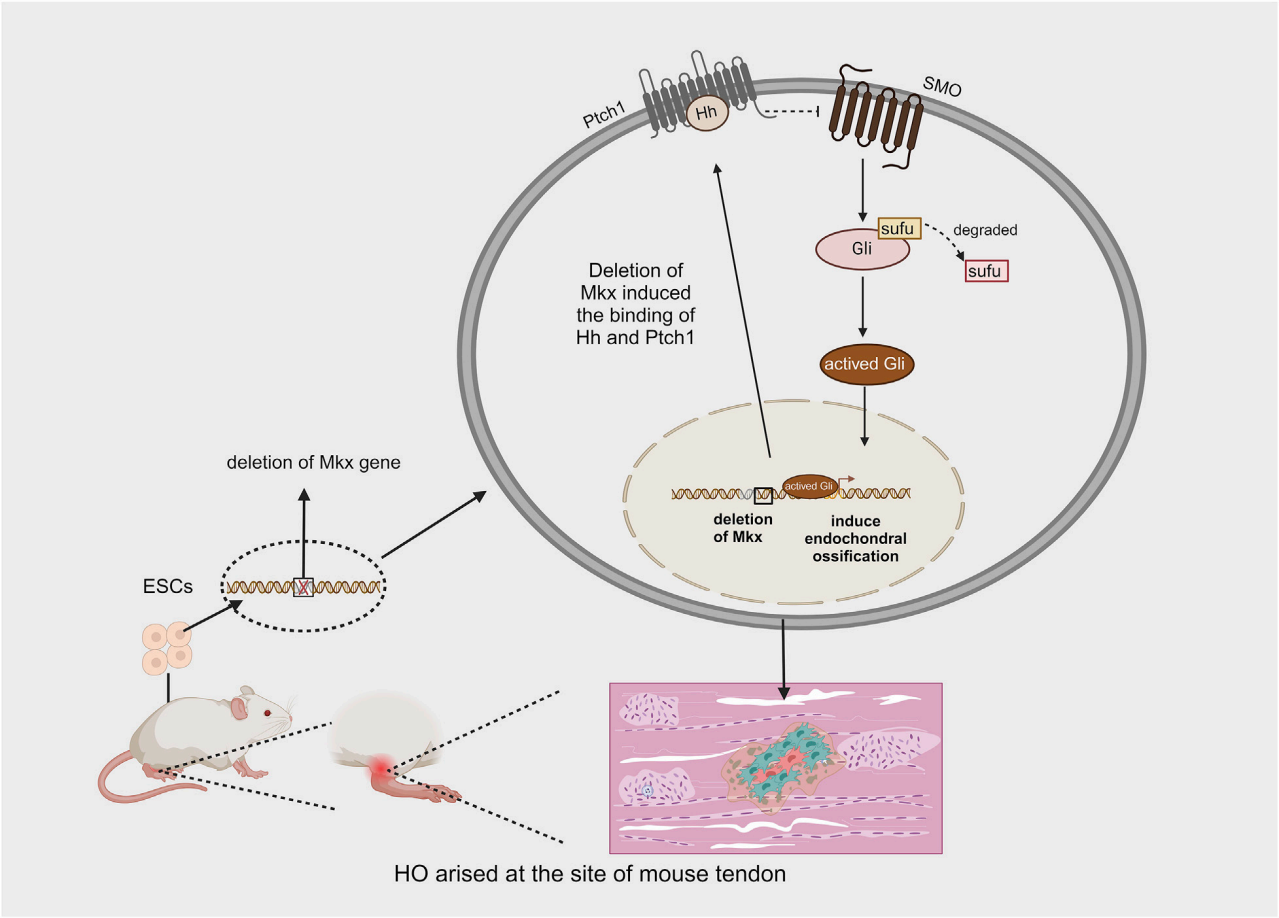
Effect of mkx deficiency in mouse embryo on the production of spontaneous HO.

The Mohawk gene (Mkx) is a member of the three amino acid loop extension (TALE) superfamily of atypical homeobox genes and is expressed during tendon development. It is responsible for promoting the maturation of various tendons. Mkx regulates the expression of type I collagen, which affects both the quantity and quality of tendon fibers via scleraxis (SCX), a basic helix-loop-helix (bHLH) transcription factor (Ito et al., 2010). Liu et al. demonstrated that the inactivation of mkx, whether before or after birth, leads to the development of ectopic ossification in the Achilles tendon of mice (Liu et al., 2019). The ectopic bone produced as a result of Mkx deficiency develops through endochondral ossification. They also observed highly activated Gli in the early stages of tendon ossification in Mkx-deficient mice, suggesting that Hh signaling is involved in the process of HO caused by Mkx deletion. To further investigate whether Hh signaling plays a key regulatory role in HO resulting from the Mkx mutation, smoothened (SMO) was knocked out in some Mkx mutant mice, leading to a significant reduction in tendon ossification in these animals. These findings demonstrate that Hh signaling, mediated by SMO, is activated following Mkx deletion and plays a critical regulatory role during ectopic tendon ossification (Liu et al., 2019) (Figure 7). Notably, the overexpression of Mkx inhibits chondrogenic and osteogenic differentiation (Suzuki et al., 2016). These findings provide new insights into the mechanisms underlying HO in tendons. However, there are several limitations to these methods: tendon ossification in mice primarily occurs in the Achilles tendon, whereas in humans, it can occur in various tendons, most commonly in the shoulder (O’Brien et al., 2002). Although both acquired and hereditary forms of HO exist in humans, acquired HO accounts for the majority of cases and typically arises after trauma, surgery, or congenital tendon disorders. Furthermore, the production of HO in Mkx-deficient rats does not depend on injury to the Achilles tendon, suggesting that the Mkx gene may be one of the genetic factors contributing to HO (Liu et al., 2019).

Research has demonstrated that ectopic ossification can occur spontaneously in the tendons of mice following the initial deletion of the Mkx gene from mouse ESCs and their subsequent culture into mature individuals. This phenomenon primarily results from the deletion of Mkx, which induces the expression of Hh signaling, ultimately activating the process of endochondral ossification (Created with BioRender.com).

## Treatment

There is currently no systematic clinical treatment for HO. Surgical intervention is an effective method for managing HO; however, considerations such as the fragility of soft tissues, the risk of post-surgical infections, slow wound healing, and potential nerve damage must be taken into account (Ranganathan et al., 2015). Pharmacological interventions have the potential to prevent HO, with NSAIDs shown to inhibit HO by suppressing osteogenic differentiation. However, an overdose of NSAIDs can be detrimental to joint healing and harmful to gastrointestinal function (Sagi et al., 2014; Burd et al., 2001). At present, no drugs are specifically approved to treat HO, although some have been discovered to inhibit HO in animal models. For instance, palovarotene, a retinoic acid receptor (RAR) agonist, can inhibit endochondral ossification in a mouse model of FOP (Holavanahalli et al., 2011). However, it remains unclear whether palovarotene also inhibits other forms of HO. Consequently, the Hh signaling pathway has been identified as a new target for HO treatment. The primary Hh signaling-related treatments involve inhibitors; however, much of the development of Hh inhibitor therapies has been focused on applications in cancer treatment, resulting in numerous clinical studies. Recently, several small molecule inhibitors have been shown to act on intermediate components of the Hh signaling pathway, regulating its expression to alleviate HO(15). For example, arsenic trioxide (ATO) is a widely used Hh signaling inhibitor for tumor therapy, which opposes the Hh pathway by targeting Gli transcriptional effectors. With extended incubation, ATO reduces the steady-state levels of Gli2, the principal activator of Hh-dependent transcription, and in the short term, it prevents Hh-induced ciliary accumulation of Gli2 (Kim et al., 2010). Additionally, ATO decreases ALP expression, the mRNA levels of transcription factors Runx2 and osterix, the expression of OCN, and the adhesion molecule vascular cell adhesion molecule-1 (VCAM-1), thereby further impeding the progression of osteogenic differentiation. However, further research is needed to determine whether ATO can be utilized in the clinical management of HO or other bone diseases (Hu et al., 2012). Many Hh inhibitors have been experimentally tested only in isolated cells and have not yet been applied to animal models; hence, they remain an emerging line of research, and it has not been established that they can be used for therapeutic purposes. For instance, BMS-833923, a SMO antagonist and Shh signaling inhibitor, has been shown to prominently suppress osteoblast differentiation in hMSCs, as evidenced by decreased ALP activity and mineralization *in vitro*, along with downregulation of osteoblast-related gene expression. qRT-PCR analysis of hMSCs treated with BMS-833923 revealed lower expression levels of Gli1 and PTCH1 compared to the control group, confirming that BMS-833923 indeed targets the Hedgehog signaling pathway (AlMuraikhi et al., 2019). GANT58, a Gli1 small molecule inhibitor, suppresses Gli1-mediated posttranslational modification to halt Gli1-dependent transcription. In these experiments, TDSCs treated with GANT58 exhibited lower ALP activity compared to control cells, and qRT-PCR analysis indicated that GANT58 treatment decreased the expression of chondrogenic markers, including aggrecan (AGG) and the α-1 chain of type II collagen (COL2A1), in TDSCs, suggesting downregulation of Hh signaling and inhibition of chondrogenic and osteogenic differentiation (Li et al., 2022). Notably, the Food and Drug Administration (FDA) has not yet approved Hh inhibitors for the treatment of bone disorders such as HO, underscoring the need for additional preclinical testing to assess the efficacy and mechanism of action of these agents.

## Perspectives

Current studies on the role of Hedgehog (Hh) signaling in the pathogenesis of heterotopic ossification (HO) are incomplete and have not fully established that Hh is entirely involved in the pathological processes of tHO and POH. Moreover, the mechanism by which Hh regulates FOP formation remains largely unknown and warrants further experimental investigation.It is also pertinent to note that Hh signaling contributes to both endochondral and intramembranous ossification in the context of HO; however, whether Hh signaling influences inflammation is still uncertain. Future research may explore the potential effects of Hh signaling on inflammation by assessing the activity of the Hh signaling pathway during the inflammatory phase in animal models undergoing HO.At this stage of research, while it has been demonstrated that other signaling molecules are involved in the regulation of HO through Hh signaling, the precise regulatory processes remain undetermined. Further exploration is necessary to ascertain whether these other signaling molecules simultaneously affect Hh in mediating HO. This could be achieved, for instance, by inactivating a specific signaling molecule and observing any subsequent impact on another signaling pathway in the mediation of HO by Hh signaling. The studies presented herein suggest that the activation of Hh signaling is not only implicated in the regulation of HO formation but may also autonomously drive HO production. Importantly, this process appears to be independent of general hereditary ectopic osteogenesis, necessitating additional studies to elucidate the specific mechanisms involved. Hh signaling has been recognized as a therapeutic target, and Hh inhibitors are currently employed in cancer therapy; however, the therapeutic effects of these inhibitors on HO remain unclear. This indicates the need for further *in vivo* experiments in animal models to validate the efficacy of these agents in treating HO. In conclusion, additional research is essential to elucidate the specific mechanisms through which Hh influences HO formation and to explore therapeutic options that target Hh signaling in the context of HO. Overall, current investigations into the role of Hh in HO formation remain incomplete, and a comprehensive understanding of the mechanisms by which Hh regulates HO is still elusive. We hope that future experiments will provide greater insight into the role of Hh in the mechanisms underlying the formation of HO.

## Conclusion

Heterotopic ossification (HO) is a pathological process characterized by the formation of ectopic bone in soft tissues. Hh signaling plays a critical role in the formation of HO, promoting its development in conjunction with other signaling molecules. It facilitates chondrogenic differentiation by interacting with Fkbp10 and PTHrP, and influences osteogenic differentiation through interactions with ERK, GNAs, and YAP. Additionally, other signaling molecules such as Runx2, BMP, and Mkx regulate both chondrogenesis and osteogenesis, with Hh signaling contributing to these processes.

## Figures and Tables

**Table:** 

**HO**	heterotopic ossification
**tHO**	traumatic heterotopic ossification
**FOP**	fibrodysplasia ossificans progressiva
**POH**	progressive osseous heteroplasia
**HEO**	Heterotopic endochondral ossification
**BMP**	bone morphogenetic protein
**ACVR1**	activator of cellular venous membrane receptor type A
**Hh**	Hedgehog
**Shh**	Sonic hedgehog
**Ihh**	Indian hedgehog
**Dhh**	Desert hedgehog
**TDPCs**	tendon-derived progenitor cells
**ROS**	reactive oxygen species
**PPIase**	peptidylprolyl isomerase
**PTHrP**	Parathyroid Hormone-Related Protein
**ERK**	Extracellular Signal-Regulated Kinase
**Cx43**	Connexin 43
**YAP**	Yes-Associated Protein
**Runx2**	Runt-Related Transcription Factor 2
**Mkx**	Mohawk gene
**TALE**	three amino acid loop extension
**SCX**	Scleraxis
**bHLH**	basic helix–loop–helix
**VCAM-1**	vascular cell adhesion molecule-1
**MSCs**	mesenchymal stem cells
**TGF-β1**	transforming growth β factor-1
**VEGF**	vascular endothelial growth factor
**PKA**	proteinase kinase A
**CK1**	casein kinase 1
**Cathepsin K**	Ctsk
**Col1α1**	type I collagen α1 chain
**OCN**	osteocalcin
**ALP**	alkaline phosphatase
**BSP**	bone silibinin
**ATO**	Arsenic trioxide
**DMSO**	Dimethyl sulfoxide
**hMSCs**	Human skeletal (mesenchymal) stem cells
**COL2A1**	α-1 chain of type II collagen
**AGG**	aggrecan
**TDSCS**	tendon-derived stem cells
**FDA**	Food and Drug Administration
